# Infective Endocarditis by *Fusobacterium* Species—A Systematic Review

**DOI:** 10.3390/pathogens14080829

**Published:** 2025-08-21

**Authors:** Petros Ioannou, Eleni Mourati, Styliani Thalasseli Kazali, Chrysoula Bali, Stella Baliou, George Samonis

**Affiliations:** 1 School of Medicine, University of Crete, 71003 Heraklion, Greece; 2 First Oncology Department, Metropolitan Hospital, Neon Faliron, 18547 Athens, Greece

**Keywords:** *Fusobacterium*, infective endocarditis, anaerobes, sepsis, embolic phenomena, dental infection

## Abstract

Background: *Fusobacterium* species are anaerobic Gram-negative bacilli that are part of the normal oropharyngeal and gastrointestinal flora. Although rare, they can cause infective endocarditis (IE), a severe condition with high morbidity. The clinical characteristics, treatment strategies, and outcomes of IE caused by *Fusobacterium* spp. remain incompletely defined. This systematic review aimed to synthesize available data on *Fusobacterium* IE and compare its features with IE caused by other pathogens. Methods: We conducted a systematic literature search in PubMed, Scopus, and the Cochrane Library up to 27 February 2025, using the terms “*Fusobacterium*” and “endocarditis.” Eligible studies included case reports or series describing adult or pediatric patients with *Fusobacterium*-associated IE. Data were extracted on demographics, risk factors, clinical features, microbiology, treatment, and outcomes. Results: A total of 21 studies (all case reports) involving 21 patients were included. The median age was 48 years, and 85.7% were male. Poor dental hygiene or recent dental work was the most common predisposing factor (47.6%). The mitral valve was most frequently affected (44.4%). Fever and sepsis were reported in nearly all cases, and embolic phenomena occurred in 81%. The most commonly isolated species were *F. necrophorum* (47.6%) and *F. nucleatum* (42.9%). Treatment commonly included metronidazole (61.9%), while surgical management was required in 23.8%. All-cause and infection-attributable mortality were both 9.5%. Conclusions: *Fusobacterium* IE predominantly affects younger males and is often linked to oral sources. This disease is associated with a high risk of systemic complications but seems to have a lower mortality compared to IE from other pathogens, including other anaerobic bacteria. Early diagnosis and appropriate antimicrobial treatment are of utmost importance for optimal outcomes. Further research is required to guide evidence-based management of this rare but serious infection.

## 1. Introduction

Infective endocarditis (IE) is a life-threatening infection that involves the endocardial surface of the heart, most frequently affecting cardiac valves, whether native or prosthetic, and increasingly involving intracardiac devices [1]. Predisposing factors for IE include structural heart disease, prosthetic valves, intravenous drug use, and recent dental or surgical procedures [2]. Despite being an uncommon condition, with an incidence estimated at up to 10 cases per 100,000 person-years, IE continues to be associated with considerable morbidity and mortality, especially in the context of delayed diagnosis or suboptimal treatment [2]. Recently, the hospital mortality for patients hospitalized with IE was 17% [3]. Another study identified the 30-day and the one-year mortality in patients with IE at 14% and 30% respectively [4].

Gram-positive bacteria, such as Staphylococci, Streptococci, and Enterococci, are the most commonly isolated microorganisms in IE, causing up to 75% of IE cases [5,6]. However, other less frequent causes of IE, such as Gram-negative microorganisms, anaerobes, and fungi, are occasionally reported [5,7,8,9]. IE by anaerobic bacteria, while rare, may be associated with more severe systemic complications and higher mortality compared to other IE pathogens. In most cases, IE due to anaerobic bacteria is caused by the anaerobic and microaerophilic streptococci, *Propionibacterium acnes*, *Bacteroides fragilis*, and *Clostridium* species [10].

*Fusobacterium* species are obligate anaerobic Gram-negative bacilli and are part of the normal flora of the gastrointestinal tract, oropharynx, and genitourinary tract [1,11]. While *Fusobacterium nucleatum* and *Fusobacterium necrophorum* are best known for their roles in periodontal disease and Lemierre’s syndrome, respectively, both have been sporadically implicated in cases of IE [1,12]. Among the species that colonize humans, *Fusobacterium nucleatum* is the most prevalent in the oral cavity and has gained significant scientific attention over the past decade due to its growing links to extraoral diseases [11]. Infective endocarditis due to *Fusobacterium* spp. is infrequent and predominantly reported in the form of isolated case reports or small case series. The clinical course can be insidious, and diagnosis is often delayed due to difficulties in culturing anaerobes and the atypical presentation of the disease [1].

Due to the scarcity of consolidated data, clinicians are often challenged in establishing optimal diagnostic and therapeutic strategies for *Fusobacterium*-associated IE. Thus, this study aims to systematically review all reported cases of IE caused by *Fusobacterium* species, and, more specifically, to describe the epidemiological, clinical, microbiological, and therapeutic characteristics of these cases and to evaluate disease outcomes, thereby providing insight into this rare but clinically significant infection.

## 2. Materials and Methods

### 2.1. Data Search

For the conduction of the present systematic review, we followed The Meta-analysis of Observational Studies in Epidemiology (MOOSE) guidelines, as they are more appropriate for systematic reviews assessing epidemiological studies (Table S1) [13]. PubMed, Cochrane Library, and Scopus were searched to identify eligible studies by using the text words: ‘*Fusobacterium* AND endocarditis’. All studies published until 27 February 2025 were included in further analysis if eligible.

### 2.2. Study Selection

The following criteria were required for inclusion of a study in the systematic review: (1) article published in the English language; (2) reporting information on microbiology, clinical characteristics, treatment, and outcomes. Exclusion criteria were the following: (1) Secondary research papers (such as reviews), editorials, and any article not providing original information on the subject; (2) studies not referring to humans; (3) studies not published in the English language; (4) Studies not referring to IE by *Fusobacterium*. One investigator (PI) used Rayyan [14] to independently review the titles and abstracts of the articles that resulted from the systematic literature search. The included studies were searched for relevant articles in their references. For articles for which the full text was not available, attempts were made to communicate with the study authors to provide the full text.

### 2.3. Outcomes of Interest

The primary scopes of the current review were to record data on: (a) the gender and age of patients with *Fusobacterium* IE and (b) the outcome of the disease. Secondary scopes were to record data on (a) the infected valve, (b) the clinical characteristics of the patients, (c) antimicrobial resistance, and (d) the treatment that was administered.

### 2.4. Data Extraction and Definitions

The present review follows the standard methodology that has been used by our study group for the review of IE in different settings [15]. The data were extracted from each eligible study by two investigators (EM, CB). Extracted data included the type of the study, the year the study was published, and the country where research was conducted; information on patient’s demographics (gender and age); the medical history of the patients (such as previous cardiac valve replacement or cardiac surgery, and time after cardiac valve replacement); data on microbiology and the infection (such as the infected valve, information regarding pathogen identification, and presence of any complications); the definitive treatment that was administered for the infection; whether patients underwent surgery along with the administration of antimicrobials, and the outcomes (such as mortality). Data on the microbiology of infection and the association of infection with mortality were recorded according to the studies’ authors. The diagnosis of IE was also confirmed by the current review’s investigators based on the data given by each study’s authors and the modified ISCVID-Dukes’ criteria if the diagnosis of IE was at least possible (presence of at least one major and one minor criterion or presence of at least three minor criteria) or if pathology established a diagnosis of IE [16]. The complications that were recorded included any clinical deterioration or organ dysfunction that was considered by each study’s authors to be associated with the IE. The quality of evidence of the included studies’ outcomes was assessed by the JBI checklist for case reports [17]. This systematic review was registered in PROSPERO (CRD420251000219. Available from https://www.crd.york.ac.uk/PROSPERO/view/CRD420251000219, accessed on 19 August 2025).

## 3. Results

### 3.1. Literature Search

**Figure 1:**
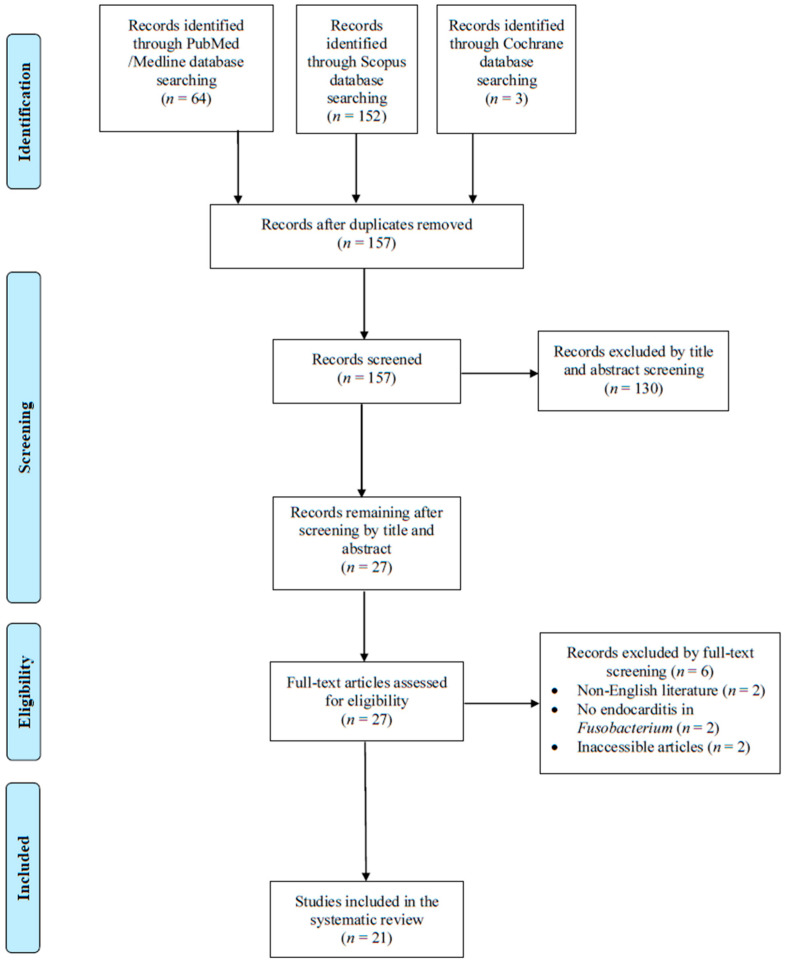
Flow diagram of study inclusion.

A total of 157 non-duplicate articles from PubMed, Cochrane Library, and Scopus were evaluated through the initial screening process. After reviewing the titles and abstracts, 27 articles were selected for review of the full text. From these studies, 6 were excluded from the review: 2 articles could not be found, 2 were not in English, and 2 were not associated with *Fusobacterium* infective endocarditis. No study was included after a search of the references of the previously mentioned studies. Finally, 21 met the inclusion criteria of the present study [18,19,20,21,22,23,24,25,26,27,28,29,30,31,32,33,34,35,36,37,38]. Figure 1 shows a graphical representation of the study inclusion procedure.

### 3.2. Included Studies’ Characteristics

The 21 studies that were eventually included in this analysis involved 21 patients. Table S2 summarizes the characteristics of the studies included. Among them, 13 were conducted in North and South America, 5 in Europe, 2 in Asia, and 1 in Africa. There were 21 case reports. The overall quality was good, as 15 articles had a low risk of bias, while 6 studies had a moderate risk of bias. The critical appraisal of the included case reports can be seen in Table S3.

### 3.3. Fusobacterium *spp.* IE Characteristics

The age of patients with *Fusobacterium* spp. IE ranged from 2 to 86 years, the median age was 48 years, the mean age was 43.9 years, and 85.7% (18 out of 21) were male. The most common predisposing factor was bad teeth hygiene or recent dental work, which was present in 47.6% (10 patients). A history of a prosthetic cardiac valve was present in 4.8% (1 patient). Table 1 shows in detail the characteristics of patients with *Fusobacterium* spp. IE.

Based on the 2023 ISCVID-Duke criteria, the diagnosis was definite in 85.7% (18 out of 21 patients), and was possible in the remaining ones. The most common sites of infection beyond the heart and the blood were the lower respiratory tract in 38.1% (8 patients), the liver in 33.3% (7), the gastrointestinal tract in 23.8% (5), the central nervous system (CNS) in 14.3% (3), and the upper respiratory tract in 4.8% (1). A concomitant infection was noted in 4.8% (1 patient) and was an abscess in the subclavian area. Infection was polymicrobial in two cases (9.5%), and the concomitantly isolated pathogens were *Staphylococcus aureus*, *Streptococcus anginosus*, *Streptococcus bovis*, *Bacteroides ruminicola* (*subsp brevis*), and *Veillonella parvula* in one patient, and *Eikenella corrodens*, *Hemophilus parainfluenzae*, *Bacteroides* spp., group C *Streptococcus*, *Corynebacterium* spp., and *Eubacterium lentum* in the other. Notably, in both cases where the infection was polymicrobial, patients were intravenous drug users. The most commonly isolated species were *F. necrophorum* in 47.6% (10 patients) and *F. nucleatum* in 42.9% (9 patients), while in two cases, the species were not defined. Antimicrobial resistance to metronidazole was 14.3% (1 out of 7 strains with available data), while to penicillin was 0% (0 out of 8 strains).

The infection was community-acquired in all cases. Fever was the most common clinical symptom and was present in all patients, while 90.5% (19 patients) had sepsis, and 15% (3 out of 20 patients) developed shock. Embolic phenomena occurred in 81% (17 out of 21 patients), heart failure was present in 23.8% (5 patients), while immunological phenomena were noted in 23.8% (5 patients).

Importantly, IE by *F. necrophorum* and IE by *F. nucleatum* seem to have slightly different characteristics, with patients suffering IE by *F. nucleatum* being of higher age, more likely to have poor dental hygiene or recent dental work, and lower mortality. However, statistical analysis was not performed due to the small number of cases.

### 3.4. Treatment and Outcome of IE by Fusobacterium *spp.*

The detailed treatment provided for *Fusobacterium* spp. IE can be seen in Table S2 and is also summarized in Table 1. The most commonly used antimicrobials were metronidazole and beta-lactams. Notably, in 42.9% (9 patients), both metronidazole and beta-lactams were used, with prolonged combination therapy used in most cases (8 out of 9 cases where the treatment was combined). Importantly, metronidazole was the only antimicrobial used for *Fusobacterium* spp. IE treatment in 3 patients. Surgical management along with antimicrobial therapy was performed in 23.8% (5 out of 21 patients). Overall, all-cause mortality was 9.5% (2 patients) and was attributed directly to IE in all patients. The median time of follow-up among survivors from the infection was 3 months (range: 0, 5–6 months).

## 4. Discussion

This systematic review presents the characteristics of patients with IE caused by *Fusobacterium* species. The results of the search strategy yielded a small number of case reports, implying that this condition is rare, while it predominantly affects middle-aged males, is often associated with poor dental hygiene or recent dental procedures as predisposing factors, and is associated with high rates of systemic complications such as sepsis and embolic phenomena. Mortality was relatively low in the present study compared to that of IE caused by other pathogens; however, the risk of severe complications remains significant, necessitating prompt recognition and appropriate management.

Compared to IE by other pathogens, in the present review, *Fusobacterium* IE patients were relatively younger. The mean age of the patients in the present review was 43.9 years for the patients in the present study versus a mean age of about 50–70 years typically reported in other IE cohorts [5,39,40,41]. Males were disproportionately affected in the present review, consistent with the general male predominance seen in IE populations [5,39,40,41]. Notably, the most frequent predisposing factor in our cohort was poor dental hygiene or recent dental intervention, aligning with the known role of the oral flora and periodontal infections [42]. In contrast, traditional IE risk factors such as prosthetic valves and congenital heart disease were rare among *Fusobacterium* IE cases, diverging from IE by typical pathogens, where these factors are prevalent [5,39,40,41].

Clinically, *Fusobacterium* IE presented with fever in all patients and sepsis in over 90%, rates higher than generally reported for IE [5,39,40,41]. Embolic phenomena were especially prominent, affecting almost 80% of patients, significantly exceeding rates seen in other types of IE, where the rate reaches 45% [5,39,41]. Despite these severe clinical presentations, the overall mortality of *Fusobacterium* IE was 9.5%, substantially lower than the >10% mortality reported for IE caused by other pathogens [5,39,41].

Anaerobic IE is generally rare and challenging to diagnose due to the fastidious nature of these organisms and limited culture sensitivity. When compared specifically with other anaerobic pathogens causing IE, such as *Bacteroides*, *Clostridium*, or *Propionibacterium* species, *Fusobacterium* IE exhibits distinct clinical and microbiological features. For example, *Clostridium* and *Clostridioides* spp. IE, that was recently reviewed by Ioannou et al., is a disease where a history of a prosthetic cardiac valve is present in one out of four patients, more commonly affects the aortic valve, but can also frequently cause complications such as sepsis and embolic phenomena [43]. In that study, one out of three patients died, contrary to the results of the present review, where IE by *Fusobacterium* spp. had a lower mortality. On the other hand, IE by *Bacteroides* spp. occurred in patients where a pre-existing heart condition was noted in more than half of the patients. Thromboembolic events were also very frequent in this type of IE, while mortality occurred in more than 30% [44]. Finally, in another example of anaerobic IE, *P. acnes* was a cause of IE in patients that almost uniformly had a prosthetic cardiac valve or an annuloplasty ring [45]. Embolic phenomena were also very common in this patient group, but mortality was very low, with only one patient out of 22 dying [45].

*Fusobacterium* spp., particularly *F. necrophorum* and *F. nucleatum*, are more frequently associated with systemic manifestations such as Lemierre’s syndrome and hepatic abscesses. Consistent with this, the present review revealed a high frequency of multi-organ involvement, including the liver (33.3%) and lungs (38.1%). This systemic dissemination appears more pronounced than in IE caused by other anaerobes, suggesting a more invasive phenotype, and maybe even more pronounced in the case of *F. necrophorum*.

Antimicrobial resistance among *Fusobacterium* species remains relatively uncommon but is increasingly reported and can pose challenges in the management of severe infections such as IE. Most clinical isolates of *Fusobacterium* necrophorum and *F. nucleatum* are susceptible to β-lactams, metronidazole, and clindamycin; however, resistance to macrolides, tetracyclines, and occasionally to metronidazole has emerged in some studies [46,47]. For example, a recent study in the United Kingdom of 313 strains of *F. necrophorum* revealed susceptibility to multiple antimicrobial agents; however, resistance to metronidazole and meropenem was detected underlying, thus, the need to investigate the antimicrobial resistance of this pathogen, and not to assume susceptibility to the recommended antimicrobials [46]. Further analyses revealed the presence of resistance genes such as the tet(M), tet(O), tet(40), ant(6)-la, aph(3′)-III, and blaOXA-85 in these *Fusobacterium* strains [46]. These findings underline the need for ongoing surveillance to detect shifts in resistance patterns, particularly given the empirical use of metronidazole in anaerobic coverage.

In terms of treatment, metronidazole was the most commonly used agent, administered in most patients, with penicillin or aminopenicillin regimens used in fewer cases. Resistance to metronidazole was observed in one case (14.3%), highlighting the need for susceptibility testing. In contrast, no resistance to penicillin was reported, underscoring its continued role in therapy for susceptible isolates. Importantly, in many patients, an antimicrobial combination of metronidazole with a beta-lactam was given. The concept of combination treatment of IE is a well-known topic that has been recently questioned, even though it still has a role for Gram-positive cocci such as *Enterococcus faecalis* and *Staphylococcus aureus* [48,49,50]. However, evidence in more rare bacteria, especially for Gram-negative ones, is scarce. Thus, it is unclear whether the combination of metronidazole with beta-lactams could provide any benefit in patients with *Fusobacterium* IE. Given the very small number of patients included in the present review and the relatively low mortality, this question could not be approached. Future multicenter studies evaluating *Fusobacterium* IE could answer whether there is a benefit from combining antimicrobials for the treatment of this disease. Surgical intervention was employed in one out of four cases, aligning with the intervention rates seen in all types of IE [5,39,40,41]. Given the high embolic risk, early surgical consultation may be warranted in the case of *Fusobacterium* IE, even in the absence of valvular destruction or heart failure.

Given the rarity but the potential severity of *Fusobacterium* IE, future multicenter studies and registry-based analyses are essential to clarify optimal diagnostic and therapeutic strategies. Enhanced awareness of this pathogen’s clinical spectrum, especially in patients with recent dental procedures or systemic *Fusobacterium* infection, could lead to an early diagnosis and improve outcomes.

This study is limited by the small number of cases and the exclusive reliance on case reports, which may introduce publication bias and limit generalizability. Moreover, there were missing data on antimicrobial susceptibilities of the strains in the studies included herein, thus hindering comprehensive analysis. Furthermore, the lack of standardized criteria for reporting anaerobic infections further complicates comparisons across studies. Additionally, due to the small number of cases, a statistical analysis between patients with IE by *F. necrophorum* and those with IE by *F. nucleatum* was not performed. Finally, some patients in the present analysis had polymicrobial infections. These studies were not excluded from the analysis; if conducted, the present systematic review would have reduced the representativeness of the real-world data about *Fusobacterium* IE. It should be noted, however, that the cases with polymicrobial infections might have different disease severity and the need for using more antimicrobials, thus affecting the outcome of the disease.

## 5. Conclusions

This systematic review highlights the epidemiological, clinical, microbiological, and therapeutic characteristics of IE caused by *Fusobacterium* species. Although rare, *Fusobacterium* IE presents with a distinct clinical profile characterized by younger age at onset, male predominance, frequent association with poor dental hygiene, and a strikingly high rate of systemic complications such as sepsis and embolic phenomena. Despite these severe manifestations, mortality remains lower than that seen in IE caused by other common or anaerobic pathogens. Given the often polymicrobial nature of the infection and the potential for extra-cardiac involvement, clinicians should maintain a high index of suspicion for *Fusobacterium* IE in patients presenting with sepsis and evidence of disseminated infection, particularly following dental procedures. Early recognition and appropriate antibiotic therapy, with consideration of surgical intervention in selected cases, are key to favorable outcomes. Further prospective studies and case registries are needed to better define the optimal diagnostic approach, antimicrobial regimen, and duration of therapy for this uncommon but serious infection.

## Figures and Tables

**Table 1:** Characteristics of patients with *Fusobacterium* spp. infective endocarditis.

Characteristic	All Patients (*n* = 21) *	*Fusobacterium* necrophorum ** (*n* = 10) *	*Fusobacterium* *nucleatum* (*n* = 9) *
Age, years, median (IQR)	48 (25–57.5)	25 (19.8–41.5)	53 (48–71.5)
Male gender, *n* (%)	18 (85.7)	8 (80)	9 (100)
Predisposing factors			
Bad teeth hygiene or recent dental work, *n* (%)	10 (47.6)	2 (20)	7 (77.8)
IVDU, *n* (%)	3 (14.3)	1 (10)	2 (22.2)
CIED, *n* (%)	2 (9.5)	0 (0)	1 (11.1)
Diabetes mellitus, *n* (%)	2 (9.5)	0 (0)	2 (22.2)
Prosthetic valve, *n* (%)	1 (4.8)	0 (0)	0 (0)
Congenital heart disease, *n* (%)	1 (4.8)	1 (10)	0 (0)
Rheumatic fever, *n* (%)	1 (4.8)	0 (0)	0 (0)
Post cardiac surgery, *n* (%)	1 (4.8)	0 (0)	0 (0)
Method of diagnosis			
Transthoracic echocardiography, *n* (%)	3/17 (17.6)	2/9 (22.2)	1/7 (14.3)
Transesophageal echocardiography, *n* (%)	13/16 (81.3)	7/9 (77.8)	5/6 (83.3)
Valve localization			
Mitral valve, *n* (%)	8/18 (44.4)	5 (50)	3/7 (42.9)
Aortic valve, *n* (%)	5/18 (27.8)	3 (30)	2/7 (28.6)
Tricuspid valve, *n* (%)	3/18 (16.7)	2 (20)	1/7 (14.3)
Right ventricle mass, *n* (%)	1/18 (5.6)	0 (0)	1/7 (14.3)
CIED, *n* (%)	1/18 (5.6)	0 (0)	0/7 (0)
Clinical characteristics			
Fever, *n* (%)	21 (100)	10 (100)	9 (100)
Sepsis, *n* (%)	19 (90.5)	10 (100)	8 (88.9)
Embolic phenomena, *n* (%)	17 (81)	7 (70)	8 (88.9)
Immunological phenomena, *n* (%)	5 (23.8)	2 (20)	2 (22.2)
Heart failure, *n* (%)	5 (23.8)	4 (40)	0 (0)
Shock, *n* (%)	3/20 (15)	2 (20)	1 (11.1)
Paravalvular abscess, *n* (%)	0 (0)	0 (0)	0 (0)
Treatment			
Metronidazole, *n* (%)	13 (61.9)	4 (40)	7 (77.8)
Aminopenicillin, *n* (%)	5 (23.8)	2 (20)	2 (22.2)
Penicillin, *n* (%)	5 (23.8)	3 (30)	2 (22.2)
Cephalosporin, *n* (%)	4 (19)	3 (30)	1 (11.1)
Carbapenem, *n* (%)	2 (9.5)	1 (10)	1 (11.1)
Antipseudomonal penicillin, *n* (%)	2 (9.5)	2 (20)	0 (0)
Clindamycin, *n* (%)	1 (4.8)	1 (10)	0 (0)
Surgical management, *n* (%)	5 (23.8)	4 (40)	0 (0)
Duration of treatment, weeks, median (IQR)	6 (6–9.8)	6 (6–9)	8.5 (4.5–13)
Outcomes			
Deaths due to infection, *n* (%)	2 (9.5)	2 (20)	0 (0)
Deaths overall, *n* (%)	2 (9.5)	2 (20)	0 (0)

CIED: cardiac implanted electronic device; IQR: interquartile range; IVDU: intravenous drug use; *: data are among the number of patients mentioned on top unless otherwise described; **: two cases of *Fusobacterium* spp. are not shown in the latter two columns but are shown in the column with all patients.

## Data Availability

Not applicable.

## Supplementary Materials

The following supporting information can be downloaded at: https://www.mdpi.com/article/10.3390/pathogens14080829/s1, Table S1: MOOSE Checklist for Meta-analyses of Observational Studies, Table S2: Characteristics of the included studies, Table S3: Risk of bias of included studies.
