# Supplementary material for: Infective Endocarditis by Fusobacterium Species—A Systematic Review
Source: Pathogens. 2025 Aug 21;14(8):829. doi: 10.3390/pathogens14080829 (PMC12389365; doi:10.3390/pathogens14080829)
Supplement: Supplementary file 1 [file pathogens-14-00829-s001.zip › Table S2.pdf]

Supplementary Table S2: Characteristics of the included studies.

Study	Number of patients	Age (years)	Gender	Diagnosis based on 2023 ISCVID-Duke criteria	Site of infection	Isolated species	Treatment administered	Infection outcome
Dhaliwal D. et al, 2023	1	86	Male	Definite	MV	*Fusobacterium nucleatum*	Aminopenicillin Sulbactam/Clavulanate	Clinical cure
M. Z. Handler et al, 2011	1	25	Male	Definite	AoV	*Fusobacterium necrophorum*	Penicillin Piperacillin/Tazobactam Surgical replacement	Clinical cure

Dahya V. et al, 2015	1	48	Male	Definite	AoV	*Fusobacterium nucleatum*	Penicillin<br>Metronidazole	Clinical cure
Sulaiman Z. I. et al, 2024	1	62	Male	Definite	AoV	*Fusobacterium nucleatum*	Cephalosporin<br>Metronidazole	Clinical cure
Galgut O. et al, 2022	1	81	Female	Possible	CIED	*Fusobacterium spp.*	Metronidazole<br>Rifampicin<br>Teicoplanin<br>(No removal of CIED)	Clinical cure

Wong E. et al, 2024	1	19	Male	Definite	MV	*Fusobacterium necrophorum*	Piperacillin/Tazobactam Metronidazole	Clinical cure
Moore C. et al, 2013	1	34	Male	Definite	MV	*Fusobacterium necrophorum*	Cephalosporin	Died
Samant J.S. et al, 2011	1	25	Male	Definite	TrV	*Fusobacterium necrophorum*	Penicillin Clindamycin	Clinical cure
G.V.L. De Socio et al, 2009	1	80	Male	Definite	MV	*Fusobacterium nucleatum*	Aminopenicillin Metronidazole	Clinical cure

Augusto J.F. et al, 2010	1	20	Female	Definite	MV	*Fusobacterium necrophorum*	Cephalosporin<br>Quinolone<br>Surgical replacement	Died
Sato K. et al, 2021	1	51	Female	Definite	MV	*Fusobacterium necrophorum*	Cephalosporin<br>Metronidazole	Clinical cure
Weber G. et al, 1999	1	37	Male	Possible	NR	*Fusobacterium nucleatum*	Carbapenem	Clinical cure
Shammas N.W. et	1	52	Male	Definite	TrV	*Fusobacterium nucleatum*	Metronidazole	Clinical cure

al, 1993								
Vedire S. et al, 2007	1	20	Male	Definite	AoV	*Fusobacterium necrophorum*	Penicillin	Clinical cure
Stuart G. et al, 1992	1	2	Male	Definite	MV	*Fusobacterium necrophorum*	Cephalosporin<br>Metronidazole<br>Vancomycin<br>Surgical replacement	Clinical cure

Goolamali S.I. et al, 2006	1	63	Male	Possible	MV	*Fusobacterium nucleatum*	Penicillin<br>Metronidazole<br>Aminoglycoside	Clinical cure
Adler A.G. et al, 1991	1	39	Male	Definite	TrV	*Fusobacterium necrophorum*<br>*Group C Streptococcus*<br>*Corynebacterium species*<br>*Eubacterium lentum*<br>*Eikenella corrodens*<br>*Hemophilus parainfluenzae*<br>*Bacteroides spp*	Aminopenicillin<br>Metronidazole<br>Vegetation & septal TrV leaflet removal	Clinical cure

Volpe N. et al, 2020	1	49	Male	Definite	AoV	*Fusobacterium necrophorum*	Aminopenicillin<br>Sulbactam/clavulanate<br>Carbapenem	Clinical cure
Storm J.C. et al, 2013	1	53	Male	Definite	Right Ventricle	*Fusobacterium nucleatum*	Metronidazole	Clinical cure
Seggie J., 1978	1	27	Male	Definite	NR	*Fusobacterium spp*	Aminopenicillin<br>Metronidazole<br>Surgical replacement of AoV & MV	Clinical cure

Levine D. et al, 1988	1	48	Male	Definite	NR	*Streptococcus anginosus* *Streptococcus bovis* *MRSA* *Fusobacterium nucleatum* *Bacteroides ruminicola* *Veillonella parvula*	Metronidazole Vancomycin Aminoglycoside	Clinical cure

AoV: aortic valve; IE: infective endocarditis CIED: cardiac implantable electronic device; MV: mitral valve; NA: not applicable; TrV: tricuspid valve
