# Supplementary material for: Infective Endocarditis by Fusobacterium Species—A Systematic Review
Source: Pathogens. 2025 Aug 21;14(8):829. doi: 10.3390/pathogens14080829 (PMC12389365; doi:10.3390/pathogens14080829)
Supplement: Supplementary file 1 [file pathogens-14-00829-s001.zip › Table S3.pdf]

Supplementary Table S3: Risk of bias of included studies

Study	Were patient's demographic characteristics clearly described?	Was the patient's history clearly described and presented as a timeline?	Was the current clinical condition of the patient on presentation clearly described?	Were diagnostic tests or assessment methods and the results clearly described?	Was the intervention(s) or treatment procedure(s) clearly described?	Was the post-intervention clinical condition clearly described?	Were adverse events (harms) or unanticipated events identified and described?	Does the case report provide takeaway lessons?	Risk of bias
Dhaliwal D. et al, 2023	Yes	Yes	Yes	Yes	Yes	Yes	Yes	Yes	Low

M. Z. Handler et al, 2011	Yes	Yes	Yes	Yes	No	Yes	No	Yes	Moderate
Dahya V. et al, 2015	Yes	Yes	Yes	Yes	Yes	Yes	Yes	No	Low
Sulaiman Z. I. et al, 2024	Yes	Yes	Yes	Yes	Yes	No	Yes	Yes	Low
Galgut O. et al, 2022	Yes	Yes	Yes	Yes	Yes	Yes	Yes	Yes	Low

Wong E. et al, 2024	Yes	Yes	Yes	Yes	Yes	Yes	Yes	Yes	Low
Moore C. et al, 2013	Yes	Yes	Yes	Yes	Yes	Yes	Yes	Yes	Low
Samant J.S. et al, 2011	Yes	Yes	Yes	Yes	Yes	Yes	Yes	Yes	Low
G.V.L. De Socio et al, 2009	Yes	Yes	Yes	Yes	Yes	Yes	Yes	Yes	Low

Augusto J.F. et al, 2010	Yes	Yes	Yes	Yes	Yes	Yes	Yes	Yes	Low
Sato K. et al, 2021	Yes	Yes	Yes	Yes	Yes	No	Yes	Yes	Low
Weber G. et al, 1999	Yes	No	Yes	Unclear	No	Yes	Yes	Yes	Moderate
Shammas N.W. et	Yes	Yes	Yes	Yes	Yes	Yes	Yes	Yes	Low

al, 1993									
Vedire S. et al, 2007	Yes	Yes	Yes	Yes	Yes	Yes	Yes	Yes	Low
Stuart G. et al, 1992	Yes	Yes	Yes	No	Yes	Yes	No	Yes	Moderate
Goolamali S.I. et al, 2006	Yes	Yes	Yes	No	Yes	Yes	Yes	Yes	Low

Adler A.G. et al, 1991	Yes	No	Yes	Yes	No	Yes	Yes	Yes	Moderate
Volpe N. et al, 2020	Yes	Yes	Yes	Yes	Yes	Yes	Yes	Yes	Low
Storm J.C. et al, 2013	Yes	Yes	Yes	Yes	Yes	Yes	Yes	Yes	Low

Seggie J., 1978	Yes	Yes	Yes	No	No	Yes	Yes	Yes	Moderate
Levine D. et al, 1988	Yes	No	Yes	Yes	Yes	Yes	No	Yes	Moderate

AoV: aortic valve; IE: infective endocarditis CIED: cardiac implantable electronic device; MV: mitral valve; NA: not applicable; TrV: tricuspid valve
